# Role of photobiomodulation in controlling the gag reflex during posterior tooth extraction, a pilot case series

**DOI:** 10.1007/s10103-026-04806-7

**Published:** 2026-01-23

**Authors:** Zeynep Çukurova Yılmaz, İpek Necla Güldiken, Alperen Tekin, Hayrunisa Koçyiğit Daştan, Buğra Düç

**Affiliations:** 1 Istanbul Medipol University, Istanbul, Turkey; 2 İstinye University, Istanbul, Turkey; 3 Istanbul Medeniyet University, Istanbul, Turkey

**Keywords:** Gagging, Low-Level light therapy, Tooth extraction, Acupuncture points

## Abstract

This pilot case series aimed to investigate the feasibility and preliminary efficacy of photobiomodulation (PBM; also known as low-level laser therapy [LLLT]) applied to specific acupuncture points (PC6 and CV24) in managing the gag reflex during posterior single-tooth extraction. Participants with moderate-to-severe gag reflex (n = 20) were selected based on Gagging Severity Index (GSI) scores ≥ 3 during clinical examination. Gag reflex severity was measured using both the GSI and the Predictive Gagging Survey (PGS) before and after the procedure. LLLT (976 nm diode laser, 100 mW, 4 J/cm² per point, continuous-wave, 20 s per point, 8-mm diameter tip [spot area ≈ 0.50 cm²]) was administered immediately prior to extraction. Preoperative GSI scores (3.3 ± 0.6) underwent a significant decrease postoperatively (1.3 ± 0.5; *P* < 0.001). Concurrently, PGS scores showed a moderate positive correlation with baseline GSI. LLLT was found to be an effective method of controlling the gag reflex during posterior tooth extraction, thereby facilitating the procedure. In this pilot study, LLLT was associated with reduced gag reflex during posterior tooth extraction. However, the absence of a control group should be acknowledged as a limitation, and the findings should be interpreted with caution. Controlled trials are needed to confirm these preliminary results.

**Supplementary Information:**

The online version contains supplementary material available at 10.1007/s10103-026-04806-7.

## Introduction

 The gag reflex is a physiological mechanism that occurs as a result of stimulation of the oropharynx, base of the tongue, and soft palate, which prevents the entry of unwanted objects into the pharynx, larynx, and trachea [1, 2]. The sensation of nausea associated with this physiological reflex is accompanied by violent spasms of the oropharyngeal muscles and a simultaneous contraction of the abdominal muscles [3, 4]. Certain factors can disrupt this physiological reflex, negatively impacting oral hygiene practices and patient cooperation during dental procedures. The gag reflex poses a significant challenge for both clinicians and patients during routine dental treatments, including intraoral radiography, third molar extractions, impression taking, denture fitting, posterior tooth preparations, and endodontic procedures. Despite its clinical importance, the exact prevalence of the gag reflex in dental settings remains unclear [5, 6].

Several methods can be used for the management of the gag reflex, including behavioral techniques, such as desensitization, relaxation, and distraction, either alone or in combination [7–9]. Nevertheless, it is important to note that these approaches may not be adequate for patients with severe gag reflexes [9]. Locally (local and topical anesthetics) or generally (antihistamines, sedatives, general anesthesia, central nervous system depressants, and parasympatholytics) acting medications are additional options for managing gag reflex; however, these medications have unwanted adverse effects [9, 10].

Acupuncture, a traditional practice dating back nearly 3000 years, involves the insertion of fine needles into specific points on the body to alleviate symptoms by modulating physiological responses, presumably via stimulation of small myelinated nerve fibers within muscle tissue, leading to subsequent activation of neural pathways including the spinal cord, midbrain, and hypothalamic-pituitary axis [11, 12]. The utilisation of acupuncture as a method to manage the intraoperative gag reflex during dental procedures has been proposed. Various acupuncture points have been recommended in the literature for this purpose. Among these, points CV24 and PC6 have been specifically identified as effective targets for controlling the gag reflex [2, 13–16]. Needle acupuncture has been reported as an effective method for managing the gag reflex [14]. With technological advancements, stimulation methods have evolved from traditional needle insertion to electroacupuncture and laser acupuncture [17]. Laser acupuncture, utilizing LLLT or photobiomodulation, offers two key advantages over needle acupuncture: it is non-invasive and thus suitable for children or patients with needle anxiety, and it significantly reduces treatment time. LLLT is a short-duration, non-invasive technique without thermal or vibrational effects, previously demonstrated to effectively suppress the gag reflex through stimulation of acupuncture points PC6 and CV24 [18, 19]. However, the efficacy of applying LLLT to these acupuncture points specifically during dental procedures for controlling the gag reflex remains unclear, particularly during posterior tooth extractions [17–19].

The primary objective of this study was to examine the efficacy of stimulating acupuncture points (PC6 and CV24) with photobiomodulation (PBM; low-level laser therapy [LLLT]) in regulating the gag reflex during posterior single-tooth extraction. To our knowledge, evidence on photobiomodulation/low-level laser therapy delivered as laser acupuncture to PC6 and CV24 immediately before posterior single-tooth extraction in an oral surgery setting is limited. This pilot case series provides initial feasibility data and preliminary effect estimates using both clinician-rated (GSI) and patient-reported (PGS) measures, which may inform the design and dosing parameters of future controlled trials.

## Methods

Patients (mean age: 43.85 ± 11.79 years) with gag reflex who visited Department of Oral and Maxillofacial Surgery for extraction of a single posterior tooth were enrolled in the present study. This study was designed as a single-arm pilot case series. The study was approved by the Ethics Committee (ethical approval no.: E-95961207-604.01.01.01–3659) and was conducted in accordance with the principles stated in the 1975 Declaration of Helsinki (as revised in 2000). Informed consent was obtained from all the patients.

The gag reflex is known to be triggered by a variety of stimuli, including sonic vibrations emanating from rotary devices, the scent and taste of dental materials, direct physical contact with the posterior regions of the mouth, exposure to dental equipment, and, in some cases, even mental imagery of dental procedures [20]. Given the lack of studies specifically addressing the relationship between jaw involvement (upper or lower) and the gag reflex during tooth extraction, the present study hypothesised that supporting the mandible with a finger inside the mouth during lower jaw tooth extraction could potentially trigger the gag reflex. Accordingly, we selected mandibular first molar extraction as a standardized posterior mandibular model, irrespective of the right/left side. All extractions were performed by the same oral surgeon under a standardized local anesthesia protocol. All included teeth were indicated for routine extraction (e.g., non-restorable caries, failed endodontic treatment, or advanced periodontal disease), and cases requiring surgical extraction were not included to minimize procedural variability.

**Fig. 1:**
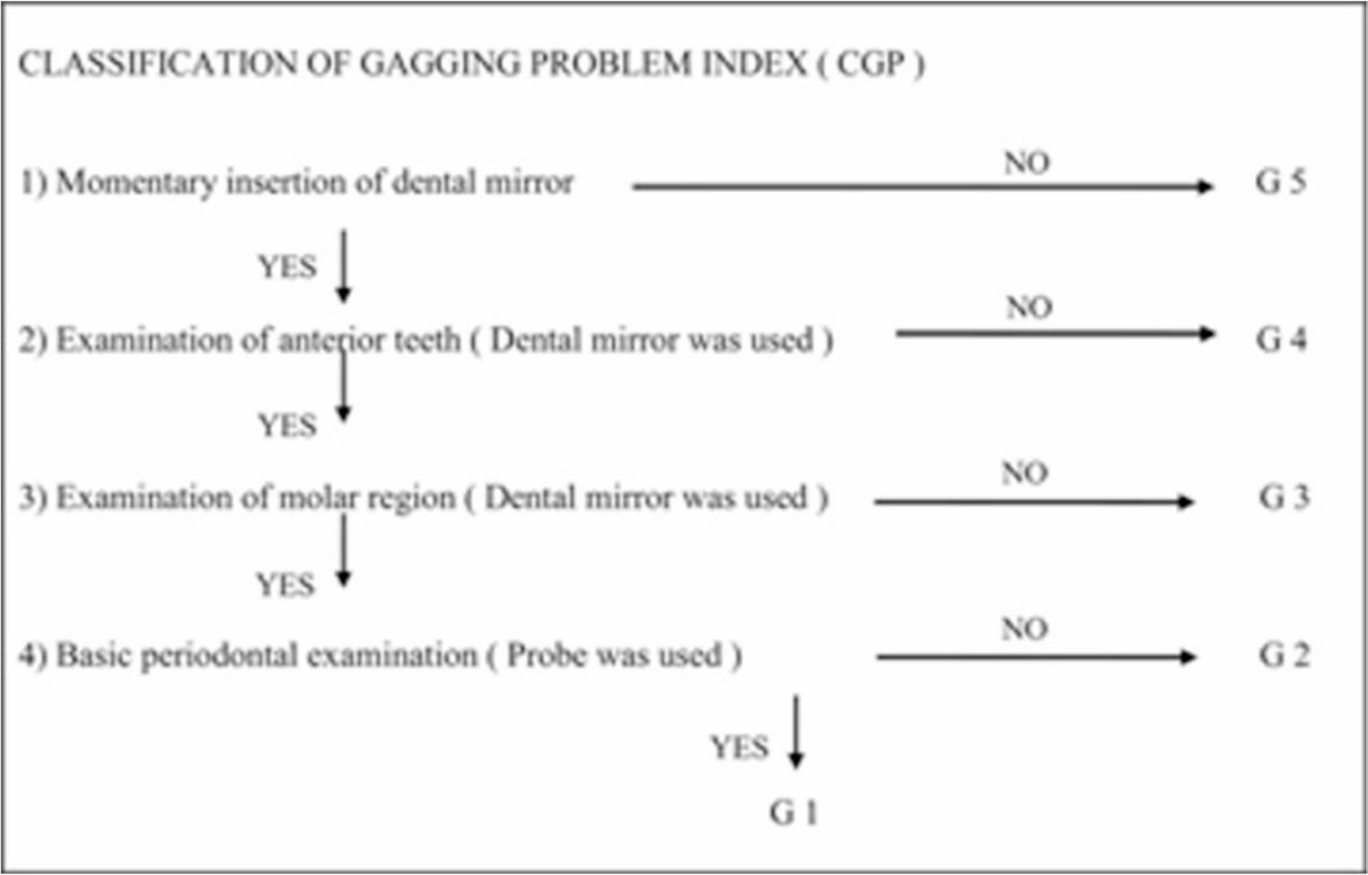
Gagging Severity Index (GSI) components

Participants were selected on the basis of the Classification of Gagging Problems Index, and those categorised as having moderate or severe gag reflexes were included in the study (Supplement 1). The severity of gagging was determined by the dentist operating the dental unit during the clinical examination using a dental mirror and probe under standard conditions with GSI (Fig. 1). The GSI is a five-point clinical scale that evaluates the intensity of the gag reflex during intraoral procedures. Despite the subjective nature of the GSI, all evaluations were conducted by the same experienced clinician under standardised conditions with a view to reducing inter-observer variability and minimising potential bias [21]. A sample size of 20 was chosen to ensure a manageable study while still providing statistically significant results. Among 34 individuals with moderate or severe gag reflexes, 20 agreed to participate in the study. Gag reflex severity was evaluated using the GSI before treatment (before the local anaesthesia administration), and measurements were repeated after LLLT [22]. A scale ranging from 1 to 5 was used for the GSI. G1, G2, G3, G4, and G5 are defined as normal, light, moderate, heavy, and very severe gagging, respectively [21].

Concurrently, the patients underwent a second test using a questionnaire. This questionnaire has been shown to accurately predict the severity of the gag reflex and exhibits high test-retest reliability (Supplement 2) [22].

The PGS is a 10-item questionnaire designed to quantify gag reflex severity through patient-reported triggers and behavioral responses. This novel tool was used to assess gag reflex intensity in all enrolled patients. The PGS was completed preoperatively by all participants (Supplement 2) [22]. The association between baseline PGS and baseline GSI was assessed using Pearson correlation. The survey scores and GSI measurements were then compared using a Pearson product-moment correlation. A copy of the questionnaire, along with scoring instructions, is provided in Supplement 2. In this survey, each answer is given a numeric score, and the overall sum indicates the severity of gagging. A total score over 7 indicates moderate gagging in response to an impression procedure.

**Fig. 2:**
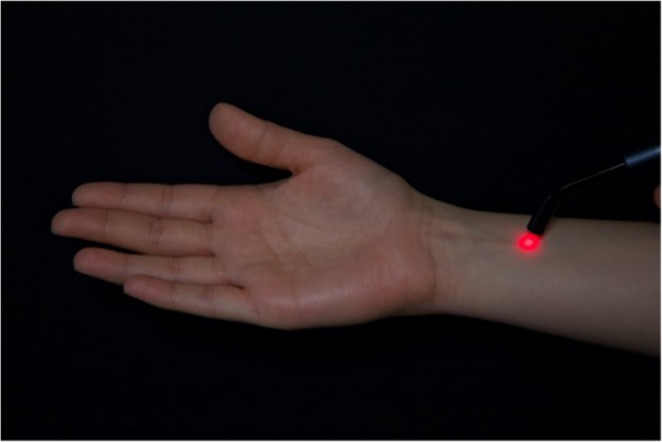
Localization of the PC6 acupoint

**Fig. 3:**
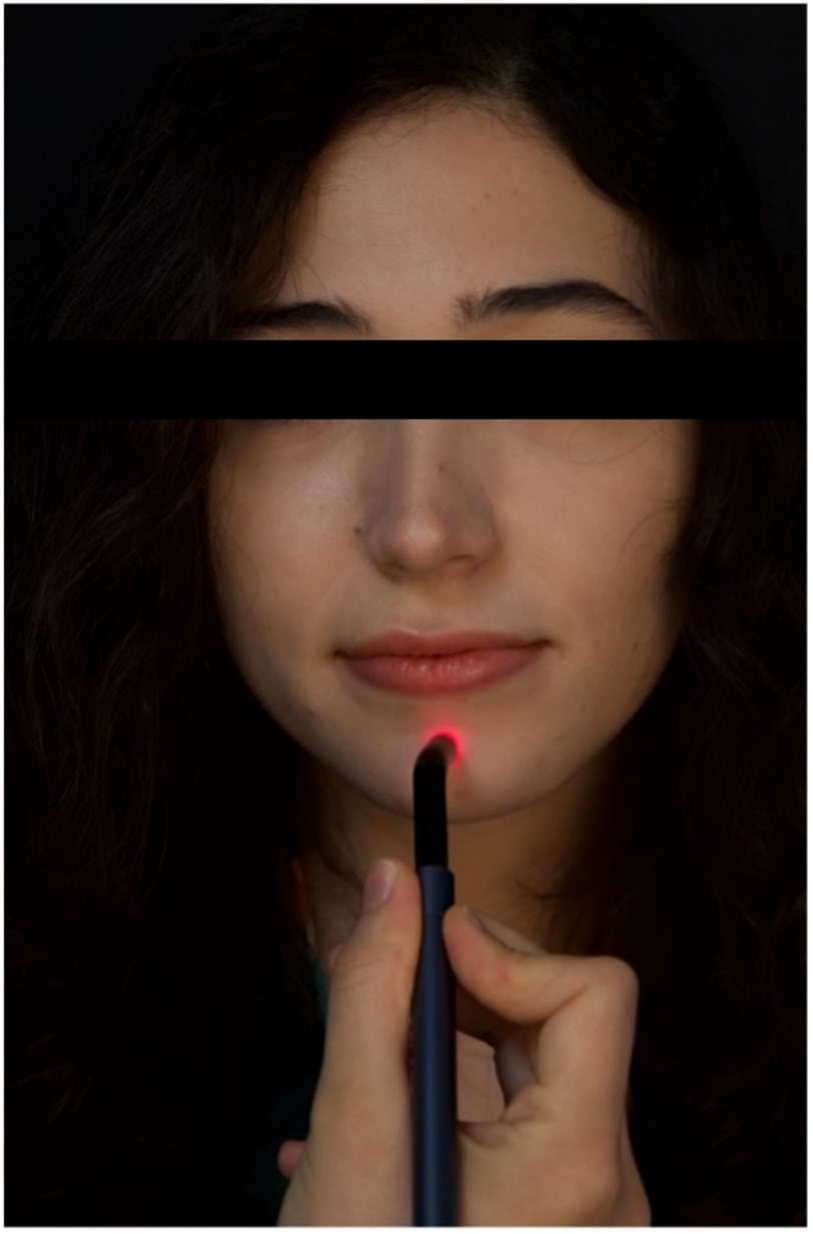
Localization of the CV24 point

LLLT was applied for 20 s per point immediately prior to tooth extraction and after local anesthesia administration to mitigate the anticipated gag reflex, targeting two acupuncture points: PC6 and CV24 [23]. The PC6 acupoint, which was identified as being located approximately three finger widths from the wrist, was stimulated for 20 s using a laser biostimulation probe. According to Usichenko et al., this point is found on the medial side of the lower arm, situated between the tendons of the *M. flexor carpi radialis* and *M. palmaris longus*, and roughly 3–4 cm above the wrist crease (Fig. 2). Concurrently, the CV24 point, located within the labiomental fold on the chin (Fig. 3), was stimulated for 20 s reinforcing the intention to alleviate the gag reflex during the dental extraction procedure [24]. A diode laser (Solase Dental Diode Laser) with a continuous wavelength of 976 nm operating in continuous mode was used with an 8-mm diameter handpiece tip (spot area ≈ 0.50 cm²). A laser probe was positioned over the target area and applied for 20 s at an output power of 100 mW (0.1 W) [23, 25–27]. The spot area (A) was calculated from the 8-mm diameter tip as A = πr², where *r* = 0.4 cm; thus A = π × (0.4 cm)² = 0.503 cm² (≈ 0.50 cm²). The delivered energy per point was E = Power × Time = 0.1 W × 20 s = 2 J. Therefore, fluence (energy density) was calculated as E/A = 2 J/0.503 cm² = 3.98 J/cm² (≈ 4 J/cm²) per point, and this dose was delivered per acupoint (PC6 and CV24). The laser was applied in non-contact mode at a distance of 2 mm from the mucosal surface.

LLLT was applied once to each acupoint (PC6 and CV24) by the same surgeon. Eye protector glasses were used by both the operator and patients. Upon completion of the procedure, the patients did not require a companion and no adverse events were observed or reported. The gag reflex scores were measured for each patient by the operator.

### Statistics

While evaluating the findings obtained in the study, Statistical Package for the Social Sciences Statistics version 22 was used for statistical analysis. The suitability of the parameters for normal distribution was evaluated using the Kolmogorov–Smirnov and Shapiro–Wilk tests. These parameters did not exhibit a normal distribution.

## Results

Twenty patients with an equal distribution of 10 males (50%) and 10 females (50%) who met the inclusion criteria were recruited. The mean age of the patients was 43.85 ± 11.79 (range, 24 − 68) years. Table 1 shows the demographic characteristics of the patients included in the study.

**Table 1:** Demographic characteristics of the patients

		*n*	%
Sex	Male	10	50
	Female	10	50
Systemic disease	No	12	60
	Yes	8	40
Drug use	No	13	65
	Yes	7	35
Smoking	No	11	55
	Yes	9	45

Evaluation of the participants’ gag reflexes according to the PGS is presented in Table 2. The mean gag reflex reported by the patients prior to the procedure was 5.3 ± 1.5. A total of 17 (85%) patients reported that they had experienced a negative incident associated with the gag reflex, whereas 18 (90%) had this reflex during treatment at the dental clinic. Tooth extraction was the procedure most frequently associated with triggering gag reflex (70%), followed by calculus removal (45%) and root canal treatment (35%) (Table 2).

**Table 2:** Evaluations of the patients regarding the gag ​​reflex

		*n*	%
GAG reflex	1*	1	5
	3	1	5
	4	1	5
	5	8	40
	6	5	25
	7	4	20
Previous negative experience of the gag reflex	No	3	15
	Yes	17	85
Experiencing a gag reflex during previous dental treatments	No	2	10
	Yes	18	90
Experiences that cause a gag reflex	Calculus removal	9	45
	Filling	3	15
	X-ray	5	25
	Root canal treatment	7	35
	Impression	6	30
	Dental extraction	14	70
	Examination	4	20
Feeling stress caused by the gag reflex on the way to the dentist’s clinic	0*	1	5
	1	3	15
	2	1	5
	3	4	20
	4	2	10
	5	3	15
	7	6	30
Daily activities such as brushing and flossing cause a gag reflex	1*	3	15
	2	6	30
	3	3	15
	4	3	15
	5	1	5
	6	2	10
	7	2	10
Daily activities other than brushing or flossing cause a gag reflex	No	6	30
	Yes	14	70
Having a gag reflex while coughing	No	14	70
	Yes	6	30
Having a gag reflex when trying to swallow medication	No	15	75
	Yes	5	25

*0: None; 7: Severe

**Fig. 4:**
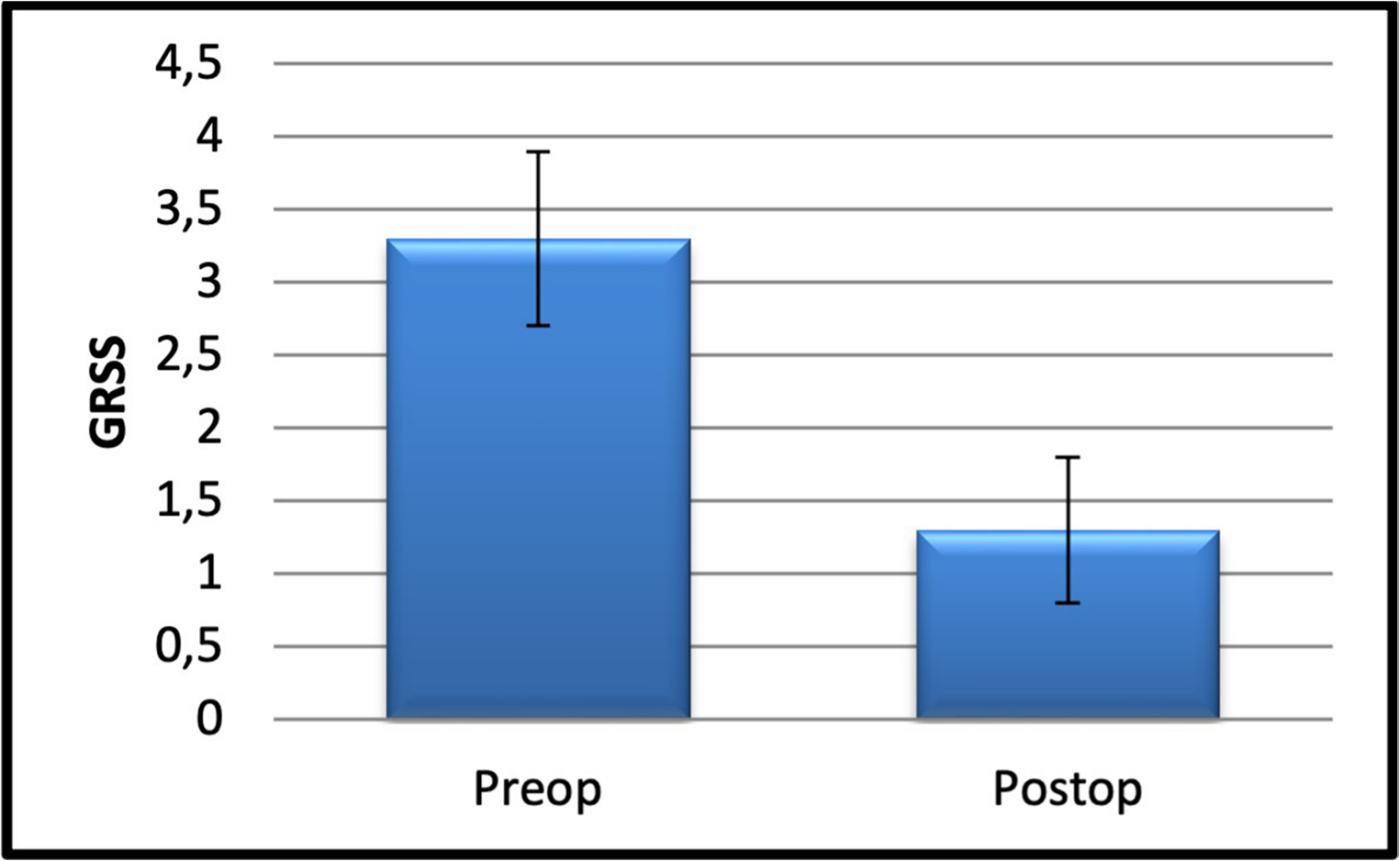
Pre- and post-treatment GSI scores

Pre- to post-procedure change. Preoperative GSI scores (3.3 ± 0.6) decreased to 1.3 ± 0.5 (Wilcoxon signed-rank, two-tailed; *P* < 0.001; Table 3; Fig. 4). Clinical interpretation: This corresponds to a mean reduction of 2.0 points (~ 60%), shifting severity from moderate (G3) toward none–light (G1–G2) and facilitating tolerability of the planned single-tooth extractions in this pilot case series. Results are also reported in Table 3 as median [IQR] and effect size (r = |z|/√n).

**Table 3:** Evaluation of the change in gag reflex severity index

GSI	Min-Max	Mean ± SD	Median	*P*
Preoperative	2–4	3.3 ± 0.6	3	< 0.001*
Postoperative	1–2	1.3 ± 0.5	1	

Wilcoxon signed-rank test (two-tailed); values reported as mean ± SD and median; α = 0.05. *P < 0.05.”

Association between PGS and baseline GSI. Self-reported gag susceptibility (PGS) showed a moderate positive association with clinician-rated baseline gagging (GSI) (Pearson, two-tailed: *r* = 0.507, *n* = 20, *P* = 0.023). The corresponding simple linear model was GSI = 0.229 × PGS + 2.087 (x = PGS, y = baseline GSI; R² ≈ 0.26), indicating that each 1-point higher PGS relates to an approximately 0.23-point higher baseline GSI.

**Fig. 5:**
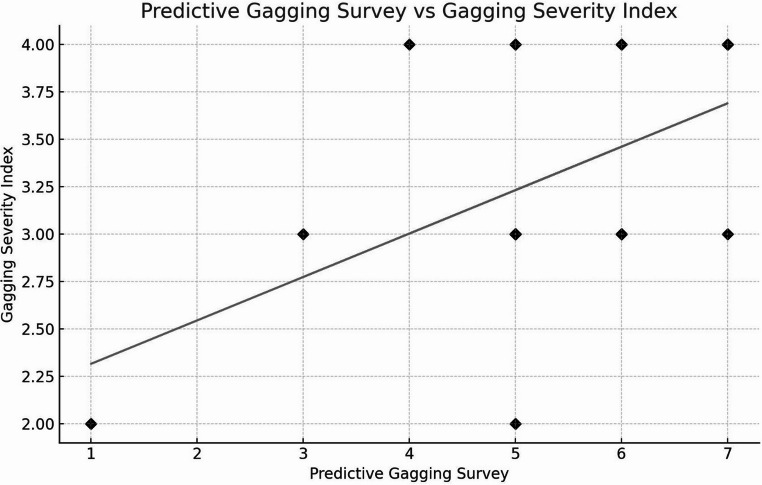
Correlation between PGS and GSI scores

The average score on the survey administered to the patients was 5.30 (standard deviation [SD] = 1.45), while the average score on the GSI was 3.30 (SD = 0.66). Scores exceeding 6 on the PGS represent the top 25% of scores (Fig. 5).

## Discussion

This study aimed to reduce the gag reflex during tooth extraction through low-level laser acupuncture, thereby facilitating the procedure for both patients and clinicians. In our study, we aimed to reduce the gag reflex in patients who underwent tooth extraction by applying low-level laser acupuncture treatment, thereby facilitating clinical management for both patients and clinicians. A pronounced gag reflex presents a significant barrier to dental care [21], frequently necessitating sedation and provoking heightened stress responses such as hypersalivation, lacrimation, and emesis. Several techniques—such as distraction, hypnosis, intravenous sedation, inhaled sedation and general anesthesia—have been employed to manage this challenging reflex. Despite the use of various techniques, no universally effective method has been established [9, 28, 29].

Acupuncture exerts its primary effect by stimulating small nerve fibers that activate the central nervous system, thereby inhibiting pain signals through a segmental gating mechanism. A secondary effect involves the stimulation of muscular trigger points, leading to vasodilation, improved oxygenation, and muscle relaxation [19]. In acupressure and laser acupuncture, specific points along energy meridians (qi or chi pathways) are stimulated without needles. MRI studies have shown that LLLT activates brain regions responsible for releasing enkephalins and endorphins, contributing to its analgesic and therapeutic effects [30]. It is well established that stimulating specific acupoints is essential for controlling the gag reflex. Research suggests that this approach can reduce nausea as effectively as, if not more than, traditional medications [31]. In the present study, we investigated whether the gag reflex could be reduced or eliminated by stimulating these acupressure points.

Moreover, PC6 (Neiguan point) and CV24 (Chengjiang point) were used as acupuncture points to control the gag reflex. Needle acupuncture is challenging to achieve in dental surgery patients (who experience pain and nervousness due to extraction), whereas laser acupuncture allows painless stimulation with easier intervention. Additionally, no side effects of LLLT have been reported in the existing literature [18].

According to Dundee and McMillan [32], PC6 stimulation with acupressure alone is ineffective in reducing postoperative vomiting in children after strabismus surgery. Evidence from a systematic review and meta-analysis suggests that PC6 stimulation can reduce postoperative vomiting in children [27]. Therefore, when developing the methodology used in the present study, laser stimulation was included. Sari and Sari combined laser stimulation of CV24 with acupressure of PC6 while taking an upper dental alginate impression in orthodontic patients and discovered that both locations had a synergistic effect in suppressing the gag reflex [29]. Similarly, in our study, the gag reflex was visibly reduced by the use of both acupuncture points during extraction. Our findings support their study using both points; however, the extraction procedure was longer than obtaining impressions. The present study is the first to compare the gag reflex before and during extraction, and the findings are encouraging.

Anatomical anomalies and neuronal hypersensitivity in the oropharynx have been recognized as somatogenic factors involved in the formation of the gag reflex [9]. In this study, the gag reflex was elicited through dental mirror insertion, with observed variations in severity serving as the basis for assessment. The findings demonstrated that the most pronounced gag reflex occurred in patients undergoing tooth extraction, followed sequentially by those receiving calculus removal and root canal treatment. It was postulated that this phenomenon may stem from oropharyngeal stimulation caused by operator-applied finger pressure to support the mandible during extraction procedures.

The questionnaire’s layout allows dentists to be aware of undesirable situations and develop techniques for managing the gag reflex through patient comments and direct inspection. In addition, the GSI helps clinicians relieve patients’ verbal and emotional comments. Strategies for detecting the reflex complaints before dental treatment have recently been established [1, 33]. It is recommended that further studies be conducted to ascertain the relationship between the GSI score and self-awareness prior to dental surgeries, with a view to facilitating more comfortable oral procedures for clinicians and patients alike. Consequently, the commencement of treatment can be initiated directly, thereby obviating the need for patient or clinician time.To the best of our knowledge, this constitutes the first quantitative comparison of pre- versus intra-operative gag reflex severity during dental extraction, yielding clinically significant outcomes.

In general, there is no standard approach for dealing with the gag reflex during dento-alveolar surgery. Any dentist should control the gag reflex based on the type of dental treatment, patient’s gag reflex level, patient’s age, and available strategies or equipment. The findings of this study indicated that LLLT constituted a feasible adjunctive approach for the patient group under consideration.

## Limitations

This study has several limitations. First, the absence of a control group prevents a definitive conclusion regarding whether the observed reduction in the gag reflex is attributable to LLLT or other confounding factors. Future studies incorporating a placebo group or alternative interventions would allow for a more robust comparison. Additionally, the small sample size limits the generalizability of the findings, highlighting the need for further investigations with larger cohorts. While this study demonstrated the short-term effects of LLLT in reducing the gag reflex, the long-term efficacy of the treatment remains uncertain. Longitudinal studies assessing whether repeated applications are necessary for sustained benefit would be particularly valuable. Furthermore, the study exclusively included patients with moderate to severe gag reflexes, thereby excluding those with mild cases and other potentially influential variables. Another important limitation is the lack of an assessment of patient anxiety levels, which could have influenced the severity of the gag reflex. Future research should incorporate validated anxiety assessment tools to explore this relationship in greater depth. Additionally, the methods used to evaluate gag reflex severity, GSI and PGS, are inherently subjective, relying on self-reports and clinician observations. While no objective measurement for gag reflex severity currently exists, future studies could benefit from the development and implementation of more standardized and reproducible assessment methods. Because the clinician who delivered LLLT also performed the post-procedure GSI assessment, detection bias is possible despite the use of predefined anchors and standardized timing/script. Additionally, the lack of blinding in the post-GSI assessment may have introduced observer (detection) bias, which should be considered a limitation of this study. A potential Hawthorne effect (behavioral change due to being observed) cannot be ruled out. Future studies will employ a blinded assessor (separate from the interventionist) within a randomized or controlled design.Finally, this study focused exclusively on single-tooth extraction in posterior mandible, a relatively short and localized dental procedure. The necessity for further research, particularly well-designed randomised controlled trials, is apparent in order to ascertain the efficacy of LLLT and to determine its applicability in more complex and protracted dento-alveolar procedures.

## Conclusions

This study provides preliminary evidence that LLLT may be effective in reducing the gag reflex during posterior tooth extractions. To the best of our knowledge, this is the first study to evaluate the gag reflex during tooth extraction within the scope of oral and maxillofacial surgery. However, given the limited sample size and the absence of a control group, the findings should be interpreted with caution. It is imperative that further randomised controlled trials are conducted, incorporating larger cohorts and comparison groups that are appropriate for the purpose. These trials are essential for confirming the clinical efficacy of LLLT in managing the gag reflex, particularly during complex dento-alveolar procedures.

## Supplementary Information

Below is the link to the electronic supplementary material.

Supplementary Material 1 (Supp-1: Classification of gag reflex severity (G1–G5)

Supplementary Material 2 (Supp-2: Predictive Gagging Survey (PGS) items)

## Data Availability

The authors confirm that the data supporting the findings of this study are available. Data generated or analyzed during this study are available from the corresponding author upon request.
