# Supplementary figures and images for: Role of photobiomodulation in controlling the gag reflex during posterior tooth extraction, a pilot case series
Source: Lasers Med Sci. 2026 Jan 23;41(1):14. doi: 10.1007/s10103-026-04806-7 (PMC12827443; doi:10.1007/s10103-026-04806-7)

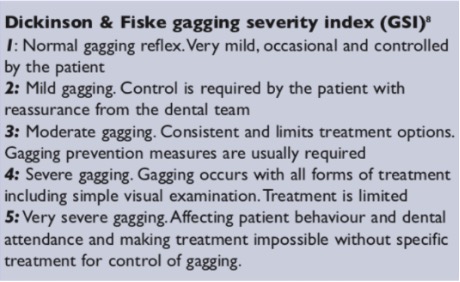

Supplement: Supplementary file 1 — Supplementary Material 1 (Supp-1: Classification of gag reflex severity (G1–G5) [file 10103_2026_4806_MOESM1_ESM.png]

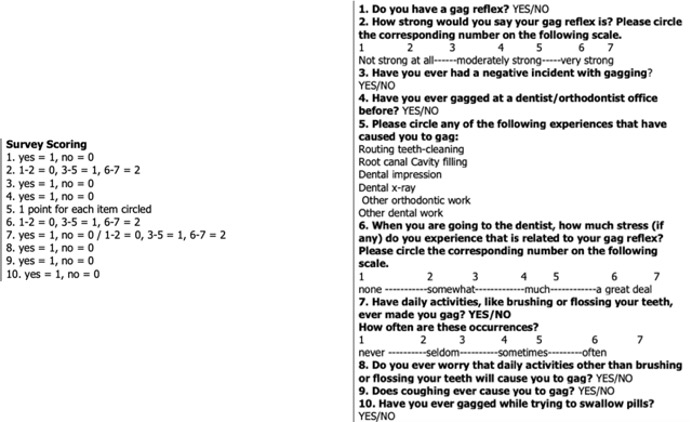

Supplement: Supplementary file 2 — Supplementary Material 2 (Supp-2: Predictive Gagging Survey (PGS) items) [file 10103_2026_4806_MOESM2_ESM.png]
